# Contrast-Induced Nephropathy in Aged Critically Ill Patients

**DOI:** 10.1155/2014/756469

**Published:** 2014-01-28

**Authors:** Eleni Palli, Demosthenes Makris, John Papanikolaou, Grigorios Garoufalis, Epaminondas Zakynthinos

**Affiliations:** Department of Critical Care, Surgical Department, University Hospital of Larissa, Biopolis, 41110 Larissa, Greece

## Abstract

*Background.* Aging is associated with renal structural changes and functional decline. The attributable risk for renal dysfunction from radiocontrast agents in critically ill older patients has not been well established. *Methods.* In this prospective study, we assessed the incidence of contrast-induced nephropathy (CIN) in critically ill patients with stable renal function who underwent computed tomography with intravenous contrast media. Patients were categorized into two age groups: <65 (YG) or ≥65 years old (OG). CIN was defined as 25% or greater increase from baseline of serum creatinine or as an absolute increase by 0.5 mg/dL until the 5th day after the infusion of contrast agent. We also evaluated the alterations in oxidative stress by assessing serum 8-isoprostane. *Results.* CIN occurred in 5 of 13 OG patients (38.46%) whereas no YG patient presented CIN (*P* = 0.015). Serum creatinine kinetics in older patients demonstrated a rise over five days following contrast infusion time while a decline was observed in the YG (*P* = 0.005). *Conclusions.* Older critically ill patients are more prone to develop renal dysfunction after the intravenous infusion of contrast agent in relation to their younger counterparts.

## 1. Introduction

Contrast-induced nephropathy (CIN) is a well-established complication of the use of the intravenous iodine contrast media representing the third most common cause of acute kidney injury in hospitalized patients [1]. The reported incidence ranges from below 5% in unselected populations to 50% in high-risk populations [2–8]. The development of CIN is associated with increase morbidity, length of hospitalization, chronic renal impairment, and higher mortality [9, 10].

Several risk factors have been related to CIN like decreased baseline renal function, heart failure, diabetes, dehydration, hypotension, older age, and the type and the amount of contrast agent applied [11–13]. Most of the studies mainly have been carried out in cardiological patients with unstable renal function who have undergone hemodynamic interventions and secondarily in other patient's groups. Critically ill is a group of patients who shared many predisposing factors for CIN which have been studied during the last few years with various results in respect to the incidence of CIN [14].

Aged critically ill patients represent a group with more compromised clinical status since kidney dysfunction is common among older people. Data from the National Health and Nutrition Examination Survey (NHANES III), a US study of community-dwelling adults, estimated that nearly 35% of the general population aged 70 years and older have moderate stage 3 chronic kidney disease [15]. Studies in Europe have also shown that there is an exponential rise in chronic kidney disease in the elderly [16–19].

In this study, we aimed to investigate whether the risk for critically ill patients >65 years old to develop CIN after exposure to intravenous contrast media is higher compared to critical care patients aged less than 65 years old. In addition, since it has been suggested that oxidative stress play a significant role in the pathogenesis mechanism of CIN, we assessed 8-isoprostane serum levels as a biomarker of oxidative stress changes following contrast infusion [20].

## 2. Material and Methods

This was a prospective observational study. Consecutive sampling was used to recruit patients from a general ICU, between 2011 and 2012.

We included consecutive critically ill patients with stable renal function who needed computed tomography (C/T) imaging with the use of intravenous contrast media during a six-month period. Exclusion criteria were unstable renal function, defined as a change in serum creatinine values greater than 0.15 mg/dL between 2 consecutive days, patients under renal replacement therapy, and history of intravascular administration of contrast agent during the 5 days before to CT scan.

The patients were divided into two groups in respect to their age: YG group included patients <65 years old and OG group which included patients ≥65 years old. Patients in the two groups were matched for APACHE II score.

### 2.1. Outcome Measures

The primary end point of the study was the incidence of CIN. Secondarily, we evaluated the alterations in oxidative stress via changes of serum levels of 8-isoprostane and the need for renal replacement therapy among the examined groups, two weeks following the infusion of the contrast agent.

### 2.2. Clinical Assessment-Definitions

We aimed to keep all participants well hydrated before and after the infusion of contrast agents; thus, 1000 mL of fluids was infused in addition to the scheduled daily requirements of each patient. The CT scans were performed with the use of low osmolarity contrast agent, Iopamiro 370, Bracco.

Serum creatinine and urea levels were measured before the administration of radiocontrast agent and thereafter once daily until the fifth day following radiocontrast infusion.

### 2.3. 8-Isoprostane Assay

Serum was sampled before the infusion of contrast agents and 24 hours later. The measurement of 8-isoprostane was performed with a commercial enzyme-linked immunoassay Kit (Cayman CC, USA).

### 2.4. Statistical Analysis

Results are expressed as means ± standard error (SE). Kolmogorov-Smirnov test was used for normality assessment. Chi-square or Fisher's exact test was used to compare categorical variables and *t*-test or Man-Whitney *U* test to compare continuous variables as appropriate. To compare serum urea and creatinine differences between subgroups over time, linear mixed model analysis was performed. Linear regression analyses were used to determine associations among continuous variables. *P* values of <0.05 were considered to be statistically significant. Statistical analysis and graphs were performed with statistical software GraphPad version 5 and the statistical package SPSS 17.0 (SPSS Inc., Chicago, IL, USA).

## 3. Results

Twenty-six critically ill patients were included in the study; 13 were <65 years old (YG) and 13 were ≥65 years old (OG).  Table 1 shows the baseline characteristics of participants; no statistically significant differences were found between groups. Two patients in the OG presented renal failure in their past medical history; both had normal diuresis and baseline serum creatinine was mildly abnormal in each one of them.

**Figure 1:**
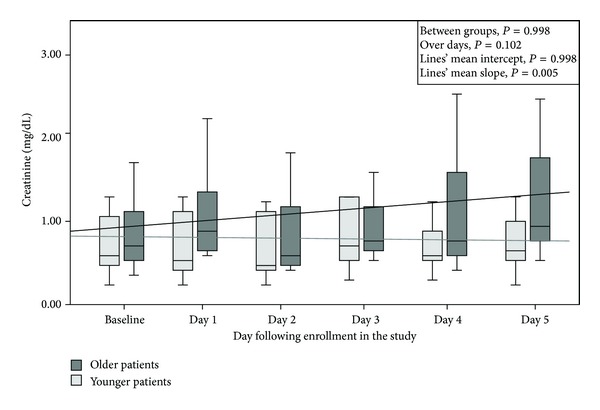
Serum creatinine (mg/dL) concentration in patients ≥65 years old and in patients <65 years old.

Five patients in the OG fulfilled the criteria for CIN 38.46%, while no one did in YG (*P* = 0.015). Serum creatinine concentration in patients of YG presented a decline over time whereas in OG there was a mild rise in serum creatinine (*P* = 0.005 for mean slope) (Figure 1). Thus, there was an indication towards higher creatinine values in the OG compared to YG at the 5th day following radiocontrast infusion (*P* = 0.07).

**Figure 2:**
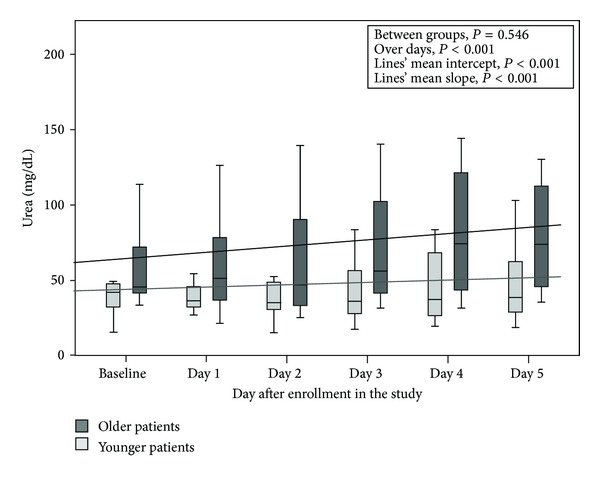
Serum urea concentrations (mg/dL) concentration in patients ≥65 years old and in patients <65 years old.

Furthermore, serum urea concentration in patients of YG presented a mild increase over time, whereas in patients of OG was detected a greater increase (*P* < 0.001 for mean slope) without significant differences between groups (*P* = 0.546) (Figure 2).

**Figure 3:**
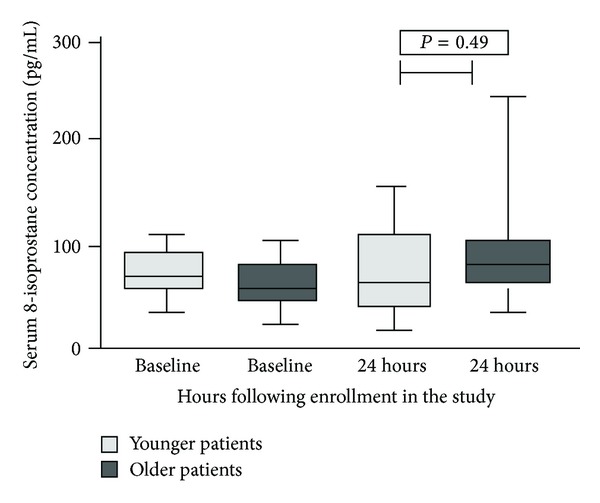
Serum levels of 8-isoprostane (pg/mL) in patients ≥65 years old and in patients <65 years old.

8-Isoprostane levels presented a peak at 24 hours after the infusion of contrasts in the OG; however, the difference compared to YG was not significant *P* = 0.49 (Figure 3).

**Figure 4:**
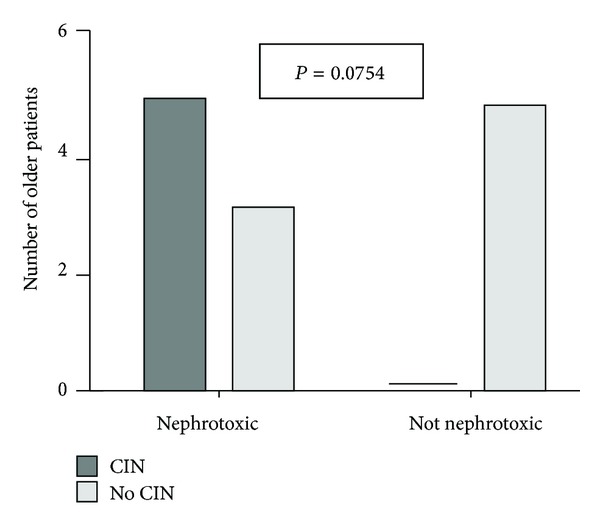
Contrast-induced nephrotoxicity (CIN) in patients ≥65 years old who developed CIN or not according to the use of nephrotoxic medications.

All OG patients who developed CIN (*n* = 5) received one at least nephrotoxic medication, four of them colimycin and one of them amikacin, while in the group of OG patients who did not develop CIN (*n* = 8), only three received a nephrotoxic medication, colimycin, *P* = 0.0754 (Figure 4). We should point out that from two patients in the OG presented renal failure in their past medical history, one of them who had also received a nephrotoxic medication developed CIN.

No difference was found between groups, in the number of patient who underwent renal replacement therapy during the two weeks following the infusion; there were 4 patients in the OG (30.8%) and one patient in the YG (7.7%) (*P* = 0.32).

## 4. Discussion

The findings of the present study suggest that critically ill patients aged 65 or more years old are more prone to present renal injury after the intravenous infusion of radiocontrast media compared to patients aged less than 65 years old. Notably, 38.46% of older patients developed CIN, but no one of the younger patients in this cohort did (*P* = 0.015). The mean serum creatinine concentration of older patients presented an estimated rise during the examined period by 0.025 mg/dL every day after the infusion of contrast agent. In contrast, serum creatinine concentration of younger patients demonstrated a slight decline during the time by 0.007 mg/dL per day after the infusion of contrast agent (Figure 1).

One might argue that older patients present significant comorbidities that may predispose to CIN. Renal function is also known to decline with age and morphological changes, such as decrease of kidney weight, appearance of sclerotic glomeruli [21], and intimal proliferation in the renal artery, are some of the causes of renal dysfunction [22]. According to the recent literature, it is not yet clear how much of the functional loss in older people is due to physiologic consequence of aging [23–25] and how much is related to associate cardiovascular disease and life course exposure to CKD risk factors such as hypertension, diabetes, and smoking [26].

A plausible hypothesis therefore for the difference in CIN between YG and OG might be the longer prevalence of chronic kidney disease, and the presence of risk factors such as hypertension, diabetes and smoking in older patients. In this cohort, however, there was no significant difference regarding several potential predisposing factors which were assessed between two groups (Table 1). Nevertheless, those factors could not be assessed precisely in our patients.

Nephrotoxic medications like nonsteroidal anti-inflammatory drugs which might have been used more in the past by older people could be also another potential explanation.

It should be also noted that there was a mild decline in serum creatinine in YG group over time which may have contributed to the differences found between YG and OG. A possible explanation for this decline in YG could be the influence of periprocedural hydration in relation to a greater reserve in the renal function of younger patients.

Regarding the special features of older patients who developed CIN in this study, an interesting observation is that all of them (*n* = 5) received one at least nephrotoxic medication, 4 of them colimycin and one of them amikacin, while in the group of older patients who did not develop CIN (*n* = 8), only 3 received a nephrotoxic medication, colimycin (*P* = 0.0754, Figure 4). Therefore, this difference could be better depicted in a larger cohort than ours and this is a limitation in our study.

In the present investigation, we used as criterion for CIN the increase in baseline serum creatinine by 25%, or the absolute increase of at least 0.5 mg/dL beyond 48 hours after the infusion of contrast agent.

This criterion is widely accepted in the literature [27]. Furthermore, the monitoring period for CIN assessment is important. In a short monitoring period, the incidence of CIN may be underestimated. Recent studies adopted longer time-periods of assessing of renal function in order to detect more cases of renal injury [28]. Our investigation is in line with this. We followed our patients for 5 days after the infusion of contrast agent. Notably, 3 patients in our study developed CIN before the 3rd day after the infusion of contrast agent while the other 2 patients at the 4th and 5ths days after, so a longer period of monitoring of renal function is considered advantageous for the diagnosis of CIN and therefore for better estimation of CIN incidence.

In the present study, we used serum creatinine as an index of renal injury. Serum creatinine concentration despite the problems related with the decreased muscle mass which is seen in older patients remains the most widely used index of renal function in clinical practice since it is a relatively inexpensive, standardized parameter which is available around the clock at almost any clinical chemistry laboratory whereas many of the new glomerular filtration rate biomarkers lack one or more of these essential features [29]. Other studies have used different indexes of renal injury such as serum cystatin C. However, results varied regarding its superiority as an indicator of renal impairment in relation to serum creatinine [29–31].

The exact pathophysiological mechanisms responsible for the development of CIN are complex and poorly understood [28]. Experimental studies suggest that the pathogenesis involves a combination of vasoconstrive free oxygen radicals generation, renal medullary ischemia, and direct tubular epithelial cell toxicity [32–34]. In this respect, we aimed to assess serum levels of 8-isoprostane as a surrogate of oxidative stress. A peak was detected 24 hours after the infusion of contrast media in OG while in YG the progress of levels represented a more constant course. 8-Isoprostane serum levels were not correlated with the alterations of serum creatinine in the same group. Between the two groups, no significant differences were found. A possible explanation is that in critically ill patients multiple medical entities which contribute to oxidative stress production may coexist; thus, their contribution might obscure the impact of radiocontrast used in CT on the oxidative burden of the critically ill patients. Certainly, one might argue that performing an additional assay like DCFH-DA might have provided further insight into the alterations of renal function due to increased oxidative stress burden. Unfortunately, we have not included an additional method for oxidative stress assessment in our study; we certainly acknowledge this limitation.

## 5. Conclusion

Older critically ill patients seem to be more susceptible in developing renal injury after the intravenous infusion of contrast agents in relation to their younger counterparts, so additional protective measures, beyond the well hydration which is the cornerstone, may have a role in prevention of CIN.

## Figures and Tables

**Table 1:** Baseline characteristics.

Characteristic	Younger patients	Older patients	*P* value
	(*n* = 13)	(*n* = 13)	
Age	44.31 ± 12.28	72.62 ± 5.18	0.0001
Male gender	13 (100%)	10 (76.92%)	0.22
Body mass index (Kg/m^2^)	24.4 ± 3.7	27.2 ± 4.1	0.59
Current smoking	5 (38.46%)	3 (23.07%)	0.67
APACHE II score in the day of CT scan	14.08 ± 4.46	17 ± 7.15	0.22
Baseline creatinine mg/dL	0.90 ± 0.66	0.89 ± 0.33	0.95
Diabetes mellitus	0	2 (15.38%)	0.48
Arterial hypertension	3 (23.07%)	6 (46.15%)	0.41
Dyslipidemia	1 (7.69%)	3 (23.07%)	0.59
Ischemic cardiac disease	1 (7.69%)	2 (15.38%)	1.0
Hepatic insufficiency	0	0	
COPD	1 (7.69%)	4 (30.76%)	0.32
Renal failure	0	2 (15.38%)	0.48
Noradrenaline *μ*g/Kg/min (*γ*)			
Low dose until ≤5*γ*	6 (46.15%)	1 (7.69%)	0.073
Medium dose until 5–20*γ*	2 (15.38%)	3 (23.76%)	1.0
High dose >20*γ*	0	1 (7.69%)	1.0
Sepsis in the last 24 h before CT scan	2 (15.38%)	3 (23.07%)	1.0
Nephrotoxic medications			
Aminoglycosides	1 (7.69%)	2 (15.38%)	1.0
Colimycin	6 (46.15%)	7 (53.84)	1.0
Teicoplanin	2 (15.38%)	3 (23.07%)	1.0
Amphotericin B	1 (7.69%)	0	1.0
ACEI	3 (23.07%)	2 (15.38%)	1.0
Diuretic	4 (30.76%)	3 (23.07%)	1.0
NSAID	4 (30.76%)	3 (23.07%)	1.0
Volume of contrast medium (mL)	100	100	1.0

COPD: chronic obstructive pulmonary disease, ACEI: angiotensin-converting-enzyme inhibitor, and NSAID: nonsteroidal antiinflammatory drugs.
